# Post-election survey data: Local democracy and the 2018 local elections in the Czech Republic

**DOI:** 10.1016/j.dib.2021.107039

**Published:** 2021-04-06

**Authors:** Pavel Šaradín, Tomáš Lebeda, Jakub Lysek, Michal Soukop, Daniela Ostrá, Eva Lebedová

**Affiliations:** Department of Politics and European Studies, Palacky University in Olomouc, Czech Republic

**Keywords:** Local politics, Social survey data, Attitudinal concepts, Sociology

## Abstract

This article presents unique survey data focused on local democracy, political attitudes and political participation. The main aim of the research was to understand the relationships between political values, political participation, political knowledge and voting behavior at the local level. The survey was held at autumn 2018 after local elections in the Czech Republic. The data are unique in terms of their focus on local politics combined with variables that are standardly examined in the context of national politics. The dataset links research fields that are directly related to local politics (local electoral participation, local electoral behavior, local non-electoral participation, trust in local institutions and evaluation of local policy efficacy) with the fields usually connected with national politics (political interest, trust in institutions, political cynicism, populist attitudes and political knowledge). Research also looks at attitudes towards democracy as such. The provided data can be used by scholars in the field of local politics, local governance and electoral behavior. Data are comparable to other large-scale individual level surveys, or may serve as data source for meta-analysis.

## Specifications Table

**Table:** 

Subject	Political science and sociology
Specific subject area	Democratic attitudes and participation at local and national levels
Type of data	Table
How data were acquired	Survey based on a researcher-made questionnaire
Data format	Raw survey data
Parameters for data collection	The data file was collected using a quota sample. The quota sample was constructed on the basis 2011 Czech population census according to: region (NUTS 3), size of place of residence, sex, age, and education. The demographic structure of respondents and its comparison with demographic structure of whole population of the Czech Republic (15+) is in section on design and methods.
Description of data collection	The questionnaire for the survey was prepared by the project team led by Pavel Šaradín and Tomáš Lebeda between 1 August and 24 September. The data collection was carried out by the Center for Public Opinion Research (CVVM) in the period after the local elections between 13 and 26 October 2018. A representative sample of 1023 respondents was selected by quota sample according to: region, size of place of residence, sex, age and education. Citizens of the Czech Republic aged 15 and over were interviewed. The interview was conducted using the PAPI (Pen-and-paper personal interview) method for 67% of respondents and the CAPI (Computer-assisted personal interview) method for 33% of respondents.
Data source location	Czech Republic
Data accessibility	Data are available in this article as supplementary files (in *CSV,* sav, and *dta* format) along with the questionnaire used in the survey. Alternatively, data can be obtained from Harvard Dataverse. Lysek, Jakub; Šaradín, Pavel, 2021, “Post-election Survey Data: Local Democracy and the 2018 Local Elections in the Czech Republic”, https://doi.org/10.7910/DVN/O7ABTH, Harvard Dataverse.

## Value of the Data

• These data are exceptional and useful because they are focusing on local governments using individual-level survey methods. The individual-level surveys are still rare regarding local politics. Insights on the patterns of local political behavior and voting can thus still be considered as a missing link (see [1], [2]). The survey was conducted as a post-election study immediately after the Czech local elections in 2018. It combines batteries of questions on concepts of political behavior, attitudes, norms, knowledge that are rarely collected in a single survey.
• The community of researchers in the field of local governance, public administration and political science will greatly benefit from the data. The data are unique in their focus on local politics combined with variables that are standardly examined in the context of national politics. This dataset links research fields that are directly related to local politics (local electoral participation, local electoral behavior, local non-electoral participation, trust in local institutions and evaluation of local policy efficacy) with fields usually connected with national politics (political interest, trust in institutions, political cynicism, populist attitudes and political knowledge etc.).
• Although they were collected in a single country, data are representative on the country level – the Czech Republic. They may be used for pooled datasets of the individual level survey across countries for comparative analysis of local governments across countries by means of hierarchical regression models and similar multivariate statistical techniques (see [3]). For example, data may be used to model the contextual effect of different country-level predictors on electoral turnout in local elections, satisfaction with local governments and trust in local governments.
• The data also served as expert material for the preparation of the government Civic Education Concept (CEC). The processing and analysis of research data thus represents a subset of specific recommendations for the preparation of the Civic Education Concept.

## 1 Data Description

We present the datafile in SPSS sav format (Supplementary File 1) and STATA 16 data format (Supplementary File 2) as well as in CSV open format. The dataset is translated into English. The SPSS and STATA format comprises all the labeling and coding that corresponds to the questionnaire. Both files can be loaded to R software (freeware) using package “haven”. In the data repository, there is a file with instructions for *R* software. The main batteries of questions were used in a standardized format as in international comparative surveys: European Social Survey (ESS), World Value Survey (WVS), Comparative Study of Electoral Systems (CSES), and International Social Survey Program (ISSP). The questionnaire and question sets were tested in a preliminary survey. The same translations as in other previous inquiries were used to word the questions. The newly formed questions were validated by experts on question construction. Additionally, we run a pilot test on a small subpopulation to ensure that the questions are not misleading for a surveyed respondent. Underlying concepts measured by batteries of questions were tested utilizing Principal Components Analysis (PCA). The questionnaire used in this survey is provided as Supplementary File 3. The data contains the following research fields.

### 1.1 Local politics

The survey was specifically aimed at local politics and citizen attitudes to local councils and local politics, including the electoral process. Regarding local politics, researchers can use this battery of questions: Did they know the candidates for the local council? Did they vote for one list of candidates or did they choose different candidates across the lists? Do they know the people elected to the local council? Does the result of the local elections mean a change in the council composition? We also asked about satisfaction with the results of the 2018 local elections and satisfaction with the activities of the previous local council.

### 1.2 Political attitudes and concepts

Previous studies have defined many political attitudes and concepts in the area of skills for democratic citizenship. However, as these attitudes and their indicators are often mixed, there is still no clarity in the field. Based on these studies, however, we tried to identify and include items in the questionnaire measuring general concepts that capture theoretically distinct sets of political attitudes.

#### 1.2.1 Institutional trust

Trust as such can be understood in terms of self-evaluation of a relationship with another entity [4]. Political trust is then defined as an evaluation of the functioning of political entities based on our own normative expectations [5]. Thus, by political trust we specifically mean the confidence that people have in political institutions as we suppose it reflects an evaluation of their performance [6]. A key position in a democratic regime is trust in political and constitutional (possibly non-constitutional) institutions and international political institutions. Respondents expressed their attitudes towards individual institutions on a traditionally used eleven-point scale with extreme values of 0 (= no trust at all) to 10 (= absolute trust). Such a generally defined scale allows respondents to determine their position more accurately, offers a higher degree of measurement accuracy and additional variance. This kind of eleven-point scale can also be used in many other different attitude measurements.

#### 1.2.2 Political interest

It can be argued that political interest simply refers to curiosity about politics aroused in citizens [7]. The question where respondents stated their interest in politics was used as one of the measurements. Additional indicators of political interest have also been added, namely frequency of discussions about politics with a partner, family, friends and colleagues. For these questions, respondents could choose from three of the following options: very often, sometimes or never.

#### 1.2.3 Following media (politics)

The concept consists of the question “how closely do you follow politics on TV (radio, newspaper or the Internet)”. A 4-point scale was used. We also used a set of questions where respondents stated how much time they spent daily … watching news and politics on TV, reading news about politics in the newspaper, listening to news about politics on the radio, reading news about politics on the Internet. In case of these questions, the respondents determined the time they spent following politics in various media using an eight-point scale.

#### 1.2.4 Political efficacy

Internal political efficacy represents a conviction about one's own ability to participate in politics, while external political efficacy refers to a perceived ability to have an influence on politics as a consequence of the responsibility of political institutions [8], [9].

#### 1.2.5 Political efficacy (internal)

Internal political efficacy was measured by five statements focused on evaluation of one's own ability to be active in politics. These statements were: “I am able to take an active role in a political group”, “I am able to participate in politics”, “I could do as good a job in a public office as most other people”, “I have a pretty good understanding of the problems facing our country”, “I am well-qualified to participate in politics”. A five-point scale was used for answering these questions.

#### 1.2.6 Political efficacy (external)

External political efficacy (alternatively referred to in the literature as political alienation) was measured with a set of statements with which respondents agree or disagree on a five-point scale. These statements were: “Politicians do not care about people”, “Politicians can be trusted”, “The main problem of our country are politicians”, “Politicians only care about the interests of the wealthy”, “Those we elect to public office lose touch with the people pretty quickly”, “Politicians are only interested in people's votes, not their opinions”, “Government does not care much what people like me think”, “It does matter who is in power”, “The people we vote for can change a lot”, “The political system allows people to have a say in what government does”, “The political system allows people to have an influence on politics”.

#### 1.2.7 Political cynicism

As defined by Cappella and Jamieson (1997:166), political cynicism means “mistrust generalized from particular leaders or political groups to the political process as a whole – a process perceived to corrupt the persons who participate in it and that draws corrupt persons as participants”. Cynicism has been defined as oppositional to political efficacy and as inversely related to trust in different social, economic, and political institutions (de Vreese 2008). To measure political cynicism, a battery of questions concerning the importance of one's vote in elections was used. Specifically, respondents indicated their agreement with claims: It is alright not to vote when a favorite party cannot win, because a lot of elections are not important. One vote in elections is negligible. Someone who does not care about the results, should not attend elections. Agreement or disagreement were determined on a five-point scale.

#### 1.2.8 Populist attitudes

This concept was measured by a set of statements: “a political compromise is just a betrayal of one's own principles”, “a strong leader in government is good for our country” and “people themselves should decide about the most important issues”. As argued in a recent study by Geurkink et al. [10], “populist attitudes tap into different latent dimensions” and are not related to concepts such as political trust and external political efficacy; each has different effects on turnout and voter's choice preferences.

#### 1.2.9 Opinion leadership

A battery of questions concerned the perception of own persuasiveness was used to measure the concept of opinion leadership (see Myers and Robertson 1972). Every respondent could determine on a five-point scale how much they agree with statements that it is they who decide about topics discussed with friends, friends discuss their topics, approve important decisions between friends, friends ask them to help them with decisions, are a source of advice for friends, are successful in persuading others, are persuasive enough so they achieve agreement during discussions, that people around act according their advice and that it is easy for them to influence others.

### 1.3 Political participation

Non-electoral political participation was a part of the survey. Respondents answered whether or not they had performed the following activities in the last 12 months: contacted a politician; worked in a political party or action group; worked in another organization or association; worn or displayed a campaign badge/sticker; signed a petition; taken part in a lawful public demonstration; boycotted certain products; posted or shared anything about politics online; visited a local council meeting; participated in free volunteering. The research was unique in that we queried for each activity whether it was related to local or national politics, both or none. As far as we know, this kind of data on political participation was collected for the first time ever.

### 1.4 Political knowledge

Research on political knowledge consists of ten statements about the political facts in the Czech Republic and international politics. Respondents were asked to answer whether the statement was true or false, but they were instructed not to guess and to use don't know in this case. The following statements were evaluated: the electoral system to the Chamber of Deputies is FPTP; The European Commission President is elected by the citizens; The Czech Republic was established in 1989; The newly established government needs a vote of confidence from both the Chamber of Deputies and the Senate; The EU has 25 member states; The regional councilors are directly elected; The region is responsible for waste management not the municipality; Canada is a permanent member of the UN Security Council; Norway is not a member of the EU. The last question concerned the name of the head of the respondent's regional council.

## 2 Experimental Design, Materials and Methods

The survey was held by the Public Opinion Research centre (CVVM) which is a division of the Institute of Sociology of the Czech Academy of Sciences. The dataset contains 154 original variables. The survey was constructed as a post-election study after the local elections 2018 (5,6 October). Generally, most of the questions were derived from international surveys ESS, ISSP, CSES and WVS to be comparable in time and with national data. The data file was collected using a quota sample. The quota sample was constructed on the basis 2011 Czech population census according to: region (NUTS 3), size of place of residence, sex, age, and education (Table).

**Table:** Demographic information of the respondents.

	Population of the Czech Republic*	Data file structure	
	*%*	*N of respondents*	*%*
**Total**	100,0	1023	100,0
**Sex**			
Men	48,8	508	49,7
Women	51,2	515	50,3
**Age**			
15–19 years	5,2	45	4,4
20–29 years old	13,5	136	13,3
30–44 years	27,7	302	29,5
45–59 years old	23,0	231	22,6
60 years or more	30,6	308	30,2
**Education**			
Elementary	13,8	132	12,9
High school without a graduation exam	33,9	350	34,3
High school with a graduation exam	33,7	346	33,9
University	18,6	193	18,9
**Size of place of residence-no of inhabitants**			
up to 799	13,6	75	7,3
800–1999	13,1	135	13,2
2000–4999	12,1	122	11,9
5000–14,999	14,0	129	12,6
15,000–29,999	10,1	170	16,7
30,000–79,999	11,5	122	11,9
80,000–999,999	13,4	134	13,1
1,000,000 or more	12,2	136	13,3
**Region – NUTS3**			
Praha	12,2	136	13,4
Středočeský	12,5	134	13,1
Jihočeský	6,0	86	8,4
Plzeňský	5,5	23	2,2
Karlovarský	2,8	35	3,4
Ústecký	7,7	116	11,3
Liberecký	4,1	35	3,4
Královehradecký	5,2	39	3,8
Pardubický	4,9	38	3,7
Vysočina	4,8	50	4,9
Jihomoravský	11,2	103	10,1
Olomoucký	6,0	45	4,4
Zlínský	5,6	74	7,2
Moravskoslezský	11,5	109	10,7

## Ethics Statement

Here we confirm that the data collection did not violated any state or EU regulations. Data are fully anonymized. All participants were fully informed and they provided a consent with anonymous data collection before questioning.

## CRediT Author Statement

**Pavel Šaradín**: Project administration, Conceptualization, Methodology; **Jakub Lysek**: Data curation, Writing - Original draft preparation; **Tomáš Lebeda**: Supervision, Methodology, Questionnaire design; **Michal Soukop:** Data curation; **Daniela Ostrá**: Data curation; **Eva Lebedová**: Reviewing and Editing.

## Declaration of Competing Interest

The authors declare that they have no known competing financial interests or personal relationships which have, or could be perceived to have, influenced the work reported in this article.

## Appendix Supplementary Materials

Image, application 1

Image, application 2

Image, application 3
