# Supplementary material for: Post-election survey data: Local democracy and the 2018 local elections in the Czech Republic
Source: Data Brief. 2021 Apr 6;36:107039. doi: 10.1016/j.dib.2021.107039 (PMC8113828; doi:10.1016/j.dib.2021.107039)
Supplement: Supplementary file 1 [file mmc1.docx]

| ***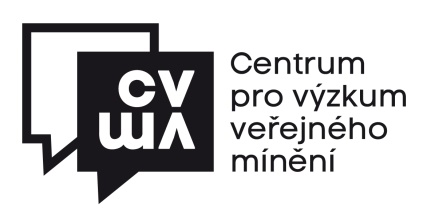*** | | | **sociologický ústav av čr, v.v.i.**  **Jilská 1**  **110 00 Praha 1** | | | | | | | | |
| --- | --- | --- | --- | --- | --- | --- | --- | --- | --- | --- | --- |
| **our society** | | | | | **13. 10. – 22. 10. 2018** | | | | **OCTOBER** | | |
|  |  |  | |  | |  |  |  | |  |  |
| coder: |  |  | | CONTROLLER: | |  |  | RECORDER: | |  |  |

| **IDE.71 Interview start time:** |  |  | **:** |  |  |
| --- | --- | --- | --- | --- | --- |

**OV.1 „„When it comes to your life, how much are you satisfied with it?“**

| You're very satisfied, .......................................................................... | 1 |  |
| --- | --- | --- |
| rather satisfied,.................................................................................. | 2 |  |
| neither satisfied nor dissatisfied,........................................................... | 3 |  |
| rather dissatisfied ,............................................................................. | 4 |  |
| very dissatisfied?“ ............................................................................ | 5 |  |
| DK ................................................................................................ | 9 |  |

PS.21 „In your opinion, what are two the most important societywide topics today?Please list them in order, according to how much they are important to you personally:"

a) ......................................................................................

b) ......................................................................................

### IDE.1 „Do you consider the standard of living of your household as

| Very good, ................................................................................ | 1 |  |
| --- | --- | --- |
| rather good, ................................................................................ | 2 |  |
| neither good nor bad, ..................................................................... | 3 |  |
| rather bad, ............................................................................... | 4 |  |
| very bad?“ ..................................................................... | 5 |  |
| DK ................................................................................................ | 9 |  |

**EV.10 „How would you evaluate the contemporary economic situation in our country? It is, in your opinion**

| Very good, ...................................................................................... | 1 |  |
| --- | --- | --- |
| good,................................................................................. | 2 |  |
| Neither good nor bad, ......................................................................... | 3 |  |
| bad,...................................................................................... | 4 |  |
| very bad?“ ................................................................................... | 5 |  |
| DK ................................................................................................ | 9 |  |

**PS.1 „Please think generally about the contemporary political situation in the Czech Republic. Would you say, you are**

| very satisfied with it, ......................................................................... | 1 |  |
| --- | --- | --- |
| rather satisfied with it , ...................................................................... | 2 |  |
| Neither satisfied, nor dissatisfied with it,............................................... | 3 |  |
| rather dissatisfied with it, ................................................................... | 4 |  |
| very dissatisfied with it?“ ................................................................... | 5 |  |
| DK ................................................................................................ | 9 |  |

***INSTRUCTION: GIVE A CARTE PI.1. TO AN INTERVIEWEE***

**PI.1 „Please tell me, do you trust**

| DEFINITELY TRUST | RATHER TRUST | RATHER DISTRUST | DEFINITELY DISTRUST | | DK |  |
| --- | --- | --- | --- | --- | --- | --- |
| 1 | 2 | 3 | 4 | | 9 |  |
| a)President of the Republic, | | | | 1 2 3 4 9 | |  |
| b) Czech government, | | | | 1 2 3 4 9 | |  |
| c) Chamber of Deputies of the Parliament of the Czech Republic | | | | 1 2 3 4 9 | |  |

| DEFINITELY TRUST | RATHER TRUST | RATHER DISTRUST | DEFINITELY DISTRUST | | DK |  |
| --- | --- | --- | --- | --- | --- | --- |
| 1 | 2 | 3 | 4 | | 9 |  |
| d) Senate of the Parliament of the Czech Republic, | | | | 1 2 3 4 9 | |  |
| e) your regional authorities, | | | | 1 2 3 4 9 | |  |
| f) your local authorities, | | | | 1 2 3 4 9 | |  |
| p) a mayor of your municipality, | | | | 1 2 3 4 9 | |  |
| q) a regional governor of your region, (mayor of Prague, if you live in Prague)? | | | | 1 2 3 4 9 | |  |

**PV.1 „„Imagine there is Parliamentary election for Chamber of Deputies next week. Would you vote?**

| Definitely yes, ...................................................... | 1 | **=> PV.4** |  |
| --- | --- | --- | --- |
| rather yes, ............................................................. | 2 | **=> PV.4** |  |
| rather not, ............................................................ | 3 | **=> PV.4** |  |
| definitely not, ....................................................... | 4 | **=> PV.37b (str. 3)** |  |
| DK?“ .......................................................... | 9 | **=> PV.4** |  |
| Not eligible to vote ............................................. | 8 | **=> PV.37b (str. 3)** |  |

***FILTER: THE FOLLOWING QUESTIONS ARE TO BE ASKED, WHEN AN INTERVIEWEE HAVE CHOSEN THE OPTIONS 1, 2, 3 OR 9 IN QUESTION PV.1 (THEY HAVE RIGHT TO VOTE AND DEFINITELY CONSIDER PARTICPATING IN ELECTION). PLEASE DO NOT FINISH WITH THE ANSWER ’I DON’T KNOW‘ AND GO FURTHER, IF POSSIBLE!***

PV.4 „If you were to vote, what party would you vote?“

............................................................................................................

**FILTER: ASK THOSE WHO IN QUESTION PV.4 DID NOT MENTION ANY PARTICULAR PARTY OR DIDN’T KNOW**

**PV.191a „Since you're not entirely sure of your choice, what parties would you consider?“**

a) ...................................................................................................

b) ...................................................................................................

***!!! ATTENTION DIFFeRENT FILTER !!!***

***FILTER: ASK THOSE WHO IN QUESTION PV.4 MENTIONED A PARTICULAR POLITICAL PARTY***

**PV.112 „How close do you feel to this party? Do you feel that you are**

| very close, .................................................................................................. | 1 |  |
| --- | --- | --- |
| pretty close, ............................................................................................... | 2 |  |
| not so close?“ ............................................................................................. | 3 |  |
| DK ............................................................................................................... | 9 |  |

PV.190 „How much are you sure that you would vote for this particular political party?

| Definitely, ..................................................... | 1 **=> PV.37b (str. 3)** |  |
| --- | --- | --- |
| rather,...................................................... | 2 **=> PV.191** |  |
| not so sure, ................................................... | 3 **=> PV.191** |  |
| not sure at all.“ ....................................... | 4 **=> PV.191** |  |
| DK .............................................................. | 9 **=> PV.191** |  |

| ***FILTER: THE FOLLOWING QUESTION SHOULD BE ASKED, WHEN IN THE QUESTION PV. 190 THE ANSWERS FROM 2 TO 4, OR 9, HAVE BEEN CHOSEN.*** |
| --- |
| **PV.191 „Assuming you are not sure who would you vote for, what other parties would you consider?“** |
| a) ................................................................................................... |
| b) ................................................................................................... |

**„And now we will focus on local election.“**

PV.37b „The council (local) elections were held on 5 and 6 October. Did you take part in the elections for your municipality or city council?

| Yes, .................................................................... | 1 **=> PV.27a** |  |
| --- | --- | --- |
| no?“ ..................................................................... | 2 **=> PV.145** |  |
| Not eligible to vote at that time.................... | 8 **=> PV.145** |  |

### *FILTER: THE QUESTIONS FOR THOSE WHO IN THE QUESTION PV. 37b HAVE CHOSEN THE OPTION 1 (THEY VOTED IN THE LOCAL ELECTION).*

**PV.27a „When did you definitely decide to vote for that particular candidate or party?**

| Just before the elections or on election day, ............................................ | 1 |  |
| --- | --- | --- |
| few days before the elections, ................................................................. | 2 |  |
| about a month before the election, ......................................................... | 3 |  |
| or earlier?“ ..................................................................................... | 4 |  |
| DK .................................................................................................... | 9 |  |

**PV.44b „Did you know the people who ran in your constituency for the local council of your municipality or city district? Did you know**

| all of them or almost all of them, ........................................................... | 1 |  |
| --- | --- | --- |
| some of them, ..................................................................................... | 2 |  |
| or did you not know any of them?“............................................................ | 3 |  |
| DK .................................................................................................... | 9 |  |

**PV.186 „In municipal elections, you have the opportunity to vote for one specific list of candidates, as well as to select candidates from several lists of candidates. Did you**

| vote for one particular list of candidates, ................................................. | 1 |  |
| --- | --- | --- |
| select candidates from more than one list of candidates?.“.......................... | 2 |  |
| DK….................................................................................................. | 9 |  |

***!!!ask all!!!***

**PV.145 „Are you satisfied with the final result of the municipal elections in your place of residence?**

| Very satisfied, ................................................................................ | 1 |  |
| --- | --- | --- |
| rather satisfied, ....................................................................................... | 2 |  |
| neither satisfied, nor dissatisfied, ......................................................... | 3 |  |
| rather dissatisfied, ............................................................................. | 4 |  |
| very dissatisfied?“ ............................................................................ | 5 |  |
| DK ...................................................................................................... | 9 |  |

PI.75 „Were you satisfied with the activities of the council of your municipality or city district and its leadership in the last election period?

| Very satisfied, ................................................................................. | 1 |  |
| --- | --- | --- |
| rather satisfied, ....................................................................................... | 2 |  |
| neither satisfied, nor dissatisfied, ............................................................ | 3 |  |
| rather dissatisfied, .................................................................................. | 4 |  |
| very dissatisfied?“ ............................................................................ | 5 |  |
| DK ..................................................................................................... | 9 |  |

PI.76 „Do you know people who were elected to the council of your municipality or city district in your constituency? Do you know

| all of them or almost all of them, ............................................................. | 1 |  |
| --- | --- | --- |
| just few of them, ................................................................................... | 2 |  |
| or don’t you know anyone?“..................................................................... | 3 |  |
| DK ...................................................................................................... | 9 |  |

PI.77 „Does the result of municipal elections in the composition of the council of your municipality or city district mean

| a big, fundamental change, .................................................................... | 1 |  |
| --- | --- | --- |
| a relatively significant change, ........................................................... | 2 |  |
| minor, not very significant change, ............................................................ | 3 |  |
| no change at all?“ .............................................................................. | 4 |  |
| DK ..................................................................................................... | 9 |  |

**PO.45a „How interested would you say you are in politics – are you:**

| Very interested, ................................................................................. | 1 |  |
| --- | --- | --- |
| rather interested, .................................................................................. | 2 |  |
| interested a little, .............................................................................. | 3 |  |
| or not at all interested?“ ........................................................................ | 4 |  |
| DK .................................................................................................... | 9 |  |

**CSN2 „And how closely do you follow politics on television, on the radio, in the newspapers or on the Internet?**

| Very closely, .................................................................................... | 1 |  |
| --- | --- | --- |
| rather closely, ................................................................................... | 2 |  |
| not so closely, .................................................................................. | 3 |  |
| or don’t you follow politics at all?“......................................................... | 4 |  |
| REFUSAL ............................................................................ | 7 |  |
| DK .................................................................................................. | 9 |  |

| ***INSTRUCTION: GIVE A CARTE TL.1 TO AN INTERVIEWEE*** | | | | | | | |
| --- | --- | --- | --- | --- | --- | --- | --- |
| **TL.1 „When you get together with your friends, relatives or fellow workers, how often do you discuss politics?** | | | | | | | |
| OFTEN | SOMETIMES | NEVER | REFUSAL | IRRELEVANT | | DK |  |
| 1 | 2 | 3 | 7 | 8 | | 9 |  |
| a) With partner, | | | | | 1 2 3 7 8 9 | |  |
| b) with other family members, | | | | | 1 2 3 7 8 9 | |  |
| c) with friends, | | | | | 1 2 3 7 8 9 | |  |
| d) with colleagues, classmates or other acquaintances?“ | | | | | 1 2 3 7 8 9 | |  |

**Q.6a „On a typical day, about how much time do you spend watching TV?**

| No time at all, ............................................. | 1 | **=> Q.6d (str. 5)** |  | |
| --- | --- | --- | --- | --- |
| less than 1 hour, ........................................... | 2 | **=> Q.6b** |  | |
| 1 hour up to 2 hours, ........................................ | 3 | **=> Q.6b** |  | |
| more than 2 hours, but no more than 3 hours, ....... | 4 | **=> Q.6b** |  | |
| more than 3 hours, but no more than 4 hours, ....... | 5 | **=> Q.6b** |  | |
| more than 4 hours, but no more than 5 hours, ....... | 6 | **=> Q.6b** |  | |
| more than 5 hours, but no more than 6 hours, ....... | 7 | **=> Q.6b** |  | |
| more than 6 hours?“ ........................................... | 8 | **=> Q.6b** |  | |
| REFUSAL......................................... | 97 | **=> Q.6d (str. 5)** |  |  |
| DK ................................................................ | 99 | **=> Q.6b** |  |  |

***FILTER: ASK ONLY THOSE WHO WATCH TV (CODES 2-8, OR 99 IN QUESTION Q.6a)***

**Q.6b „And how much of this time do you spend watching news about politics and current affairs?**

| No time at all, ............................................................................ | 1 |  |  | |
| --- | --- | --- | --- | --- |
| less than ½ hour, ....................................................................... | 2 |  |  | |
| ½ hour up to 1 hour, .................................................................... | 3 |  |  | |
| more than 1 hour, but no more than 1,5 hours, ................................. | 4 |  |  | |
| more than 1,5 hours, but no more than 2 hours, ............................... | 5 |  |  | |
| more than 2 hours, but no more than 2,5 hours, ................................ | 6 |  |  | |
| more than 2,5 hours, but no more than 3 hours, .............................. | 7 |  |  | |
| more than 3 hours?“ .................................................................... | 8 |  |  | |
| REFUSAL...................................................................... | 97 |  |  |  |
| DK ............................................................................................. | 99 |  |  |  |

***!!!ASK ALL!!!***

**Q.6d „On a typical day, about how much time do you spend reading printed newspaper?**

| No time at all, ............................................................ | 1 | **=> Q.6g** |  | |
| --- | --- | --- | --- | --- |
| less than ½ hour, ........................................................ | 2 | **=> Q.6e** |  | |
| ½ hour up to 1 hour, ..................................................... | 3 | **=> Q.6e** |  | |
| more than 1 hour, but no more than 1,5 hours, .................. | 4 | **=> Q.6e** |  | |
| more than 1,5 hours, but no more than 2 hours, ............... | 5 | **=> Q.6e** |  | |
| more than 2 hours, but no more than 2,5 hours, .............. | 6 | **=> Q.6e** |  | |
| more than 2,5 hours, but no more than 3 hours, ............... | 7 | **=> Q.6e** |  | |
| more than 3 hours?“ ..................................................... | 8 | **=> Q.6e** |  | |
| REFUSAL...................................................... | 97 | **=> Q.6g** |  |  |
| DK ............................................................................. | 99 | **=> Q.6e** |  |  |

***FILTER: ASK ONLY THOSE WHO READ NEWSPAPERS (CODES 2-8, OR 99 IN QUESTION Q.6d)***

**Q.6e „And how much of this time do you spend reading news about politics and current affairs ?**

| No time at all, ............................................................................. | 1 |  | |
| --- | --- | --- | --- |
| less than ½ hour, ......................................................................... | 2 |  | |
| ½ hour up to 1 hour,....................................................................... | 3 |  | |
| more than 1 hour, but no more than 1,5 hours, .................................. | 4 |  | |
| more than 1,5 hours, but no more than 2 hours, ................................. | 5 |  | |
| more than 2 hours, but no more than 2,5 hours, .................................. | 6 |  | |
| more than 2,5 hours, but no more than 3 hours, .................................. | 7 |  | |
| more than 3 hours?“ ...................................................................... | 8 |  | |
| REFUSAL........................................................................ | 97 |  |  |
| DK .............................................................................................. | 99 |  |  |

***!!!ask all!!!***

**Q.6g „On a typical day, about how much time do you spend listening to the radio?**

| No time at all, ............................................... | 1 | **=> Q.6i (str.6)** |  | |
| --- | --- | --- | --- | --- |
| less than ½ hour, ............................................ | 2 | **=> Q.6h** |  | |
| 1 hour up to 2 hours, ......................................... | 3 | **=> Q.6h** |  | |
| more than 2 hours, but no more than 3 hours, ...... | 4 | **=> Q.6h** |  | |
| more than 3 hours, but no more than 4 hours, ...... | 5 | **=> Q.6h** |  | |
| more than 4 hours, but no more than 5 hours,....... | 6 | **=> Q.6h** |  | |
| more than 5 hours, but no more than 6 hours, ....... | 7 | **=> Q.6h** |  | |
| more than 6 hours?“ ......................................... | 8 | **=> Q.6h** |  | |
| REFUSAL.......................................... | 97 | **=> Q.6i (str.6)** |  |  |
| DK ................................................................ | 99 | **=> Q.6h** |  |  |

***FILTR: ASK ONLY THOSE WHO LISTEN TO THE RADIO (CODES 2-8, OR 99 IN QUESTION Q.6g)***

**Q.6h „And how much of this time do you spend listening to the news about politics and current affairs**

| No time at all, .............................................................................. | 1 |  | |
| --- | --- | --- | --- |
| less than ½ hour, ........................................................................... | 2 |  | |
| ½ hour up to 1 hour, ........................................................................ | 3 |  | |
| more than 1 hour, but no more than 1,5 hours, .................................... | 4 |  | |
| more than 1,5 hours, but no more than 2 hours, .................................. | 5 |  | |
| more than 2 hours, but no more than 2,5 hours, ................................... | 6 |  | |
| more than 2,5 hours, but no more than 3 hours, ................................ | 7 |  | |
| more than 3 hours?“ ........................................................................ | 8 |  | |
| REFUSAL ......................................................................... | 97 |  |  |
| DK ................................................................................................ | 99 |  |  |

***!!!ask all!!!***

**Q.6i „On a typical day, about how much time do you spend using the internet just for personal use?**

| No time at all,.............................................................. | 1 | **=> B.2** |  | |
| --- | --- | --- | --- | --- |
| less than ½ hour, ......................................................... | 2 | **=> Q.6j** |  | |
| ½ hour up to one hour, .................................................... | 3 | **=> Q.6j** |  | |
| more than 1 hour, but no more than 1.5 hours, .................... | 4 | **=> Q.6j** |  | |
| more than 1,5 hours, but no more than 2 hours,.................. | 5 | **=> Q.6j** |  | |
| more than 2 hours, but no more than 2,5 hours, .................. | 6 | **=> Q.6j** |  | |
| more than 2,5 hours, but no more than 3 hours, .................. | 7 | **=> Q.6j** |  | |
| more than 3 hours?“ ....................................................... | 8 | **=> Q.6j** |  | |
| REFUSAL........................................................ | 97 | **=> B.2** |  |  |
| DK .............................................................................. | 99 | **=> Q.6j** |  |  |

***FILTER: ASK ONLY THOSE WHO CHOOSED OPTION 2-8 OR 99 IN THE PREVIOUS QUESTION Q.6i.***

**Q.6j „And how much of your time spend online is devoted to reading news about politics and current events??**

| No time at all, .............................................................................. | 1 |  | |
| --- | --- | --- | --- |
| less than ½ hour, ........................................................................... | 2 |  | |
| ½ hour up to one hour, ....................................................................... | 3 |  | |
| more than 1 hour, but no more than 1.5 hours, .................................... | 4 |  | |
| more than 1,5 hours, but no more than 2 hours, .................................... | 5 |  | |
| more than 2 hours, but no more than 2,5 hours, .................................... | 6 |  | |
| more than 2,5 hours, but no more than 3 hours, ................................... | 7 |  | |
| more than 3 hours?“ ......................................................................... | 8 |  | |
| REFUSAL.......................................................................... | 97 |  |  |
| DK ................................................................................................ | 99 |  |  |

***!!!ask all!!!***

| **B.2 „How much would you say the political system in the Czech Republic allows people like you to have a say in what the government does?** | | |
| --- | --- | --- |
| Not at all,.......................................................................................... | 1 |  |
| very little, ...................................................................................... | 2 |  |
| some,.............................................................................................. | 3 |  |
| a lot,..................................................................................... | 4 |  |
| a great deal.“................................................................................ | 5 |  |
| REFUSAL.......................................................................... | 7 |  |
| DK................................................................................................. | 9 |  |

| **B.3 „How able do you think you are to take an active role in a group involved with political issues?** | | |
| --- | --- | --- |
| Not at all able,............................................................................. | 1 |  |
| a little able, ................................................................................ | 2 |  |
| quite able,................................................................................ | 3 |  |
| very able,.................................................................................. | 4 |  |
| completely able.“............................................................................ | 5 |  |
| REFUSAL......................................................................... | 7 |  |
| DK................................................................................................ | 9 |  |

| **B.4 „How much would you say the political system in our country allows people like you to have an influence on politics?** | | |
| --- | --- | --- |
| Not at all,......................................................................................... | 1 |  |
| very little, ...................................................................................... | 2 |  |
| some,............................................................................................. | 3 |  |
| a lot,..................................................................................... | 4 |  |
| a great deal.“................................................................................ | 5 |  |
| REFUSAL......................................................................... | 7 |  |
| DK................................................................................................ | 9 |  |

| **B.5 „How confident are you in your own abillity to participate in politics?** | | |
| --- | --- | --- |
| Not at all confident,........................................................................... | 1 |  |
| a little confident, .............................................................................. | 2 |  |
| quite confident,................................................................................ | 3 |  |
| very confident,................................................................................. | 4 |  |
| completely confident.“....................................................................... | 5 |  |
| REFUSAL......................................................................... | 7 |  |
| DK................................................................................................ | 9 |  |

| ***INSTRUCTION: GIVE A CARTE TL.2* *TO AN INTERVIEWEE*** | | | | | | | | | | | | | | | | | | | | |
| --- | --- | --- | --- | --- | --- | --- | --- | --- | --- | --- | --- | --- | --- | --- | --- | --- | --- | --- | --- | --- |
| **TL.2 „Using this card, please tell me on a score of 0-10 how much you personally trust each of the institutions I read out. 0 means you do not trust an institution at all, and 10 means you have complete trust. Firstly...** | | | | | | | | | | | | | | | | | | | | |
| NO TRUST AT ALL | | | |  | |  | |  | |  | |  | | | complete trust | | | REFUSAL | DK |  |
| 0 | 1 | 2 | 3 | | 4 | | 5 | | 6 | | 7 | | | 8 | | 9 | 10 | 97 | 99 |  |
| a)the Czech parliament, | | | | | | | | | | | | | 0 1 2 3 4 5 6 7 8 9 10 97 99 | | | | | | |  |
| b)the legal system, | | | | | | | | | | | | | 0 1 2 3 4 5 6 7 8 9 10 97 99 | | | | | | |  |
| c)the police, | | | | | | | | | | | | | 0 1 2 3 4 5 6 7 8 9 10 97 99 | | | | | | |  |
| d) politicians, | | | | | | | | | | | | | 0 1 2 3 4 5 6 7 8 9 10 97 99 | | | | | | |  |
| e) political parties, | | | | | | | | | | | | | 0 1 2 3 4 5 6 7 8 9 10 97 99 | | | | | | |  |
| f)the European Parliament, | | | | | | | | | | | | | 0 1 2 3 4 5 6 7 8 9 10 97 99 | | | | | | |  |
| g) media, | | | | | | | | | | | | | 0 1 2 3 4 5 6 7 8 9 10 97 99 | | | | | | |  |
| h) the United Nations?“ | | | | | | | | | | | | | 0 1 2 3 4 5 6 7 8 9 10 97 99 | | | | | | |  |

| ***INSTRUCTION: GIVE A CARTE TL.3 TO AN INTERVIEWEE*** | | | | | | | | |
| --- | --- | --- | --- | --- | --- | --- | --- | --- |
| **TL.3 There are different ways of trying to improve things in Czech Republic or help prevent things from going wrong. During the last 12 months, have you done any of the following? If so, tell us if it related to matters in your municipality or to wider ones, e.g. national ones.** | | | | | | | | |
| YES, IN MATTERS RELATING TO THE MUNICIPALITY | YES, IN THE WIDER ONES | YES, IN BOTH PREVIOUS MATTERS | NO | REFUSAL | | DK |  |  |
| 1 | 2 | 3 | 4 | 7 | | 9 |  |  |
| 1. contacted a politician, government or local government official | | | | | 1 2 3 4 7 9 | |  | |
| b) worked in a political party or action group, | | | | | 1 2 3 4 7 9 | |  | |
| c) worked in another organisation or association, | | | | | 1 2 3 4 7 9 | |  | |
| d) worn or displayed a campaign badge/sticker, | | | | | 1 2 3 4 7 9 | |  | |
| e) signed a petition, | | | | | 1 2 3 4 7 9 | |  | |
| f) taken part in a lawful public demonstration, | | | | | 1 2 3 4 7 9 | |  | |
| g) boycotted certain products, | | | | | 1 2 3 4 7 9 | |  | |
| h) posted or shared anything about politics online, for example on blogs, via email or on social media such as Facebook or Twitter | | | | | 1 2 3 4 7 9 | |  | |
| i) engaged in illegal protest activities, | | | | | 1 2 3 4 7 9 | |  | |
| j) participated in free volunteer work, | | | | | 1 2 3 4 7 9 | |  | |
| k) visited the proceedings of any elected body, parliament or council | | | | | 1 2 3 4 7 9 | |  | |
| l) attended an educational course or similar event aimed at  to expand civic awareness or political knowledge?“ | | | | | 1 2 3 4 7 9 | |  | |

***INSTRUCTION: GIVE A CARTE CSN4 TO AN INTERVIEWEE***

**CSN4 „To what extent do you agree or disagree with each of the following statements“?**

| AGREE STRONGLY | AGREE | NEITHER AGREE NOR DISAGREE | DISAGREE | DISAGREE STRONGLY | | REFUSAL | DK |  |
| --- | --- | --- | --- | --- | --- | --- | --- | --- |
| 1 | 2 | 3 | 4 | 5 | | 7 | 9 |  |
| a) What is commonly referred to in politics as compromise is just a betrayal of one's own principles. | | | | | 1 2 3 4 5 7 9 | | |  |
| b) Most politicians don’t care about people. | | | | | 1 2 3 4 5 7 9 | | |  |
| c)Most politicians can be trusted. | | | | | 1 2 3 4 5 7 9 | | |  |
| d) The main problem in the Czech Republic is its politicians. | | | | | 1 2 3 4 5 7 9 | | |  |
| e) Having a strong leader in government is good for our country, even if that leader is bending the rules to get things done. | | | | | 1 2 3 4 5 7 9 | | |  |
| f) The most important political issues should be decided by the people, not by politicians. | | | | | 1 2 3 4 5 7 9 | | |  |
| g) Most politicians only care about the interests of the rich and powerful.“ | | | | | 1 2 3 4 5 7 9 | | |  |

***INSTRUCTION: GIVE A CARTE Q.14 TO AN INTERVIEWEE***

**Q.14 „Some believe it doesn't matter who's in power at all. Others**

**think it matters a lot. If you were to use the scale on this card (where ONE means no matter who is in power, and FIVE means that who is in power matters very much), where would you place your opinion?“**

| NO MATTER WHO IS IN POWER | | | WHO IS IN POWER MATTERS VERY MUCH | | | REFUSAL | DK |  |
| --- | --- | --- | --- | --- | --- | --- | --- | --- |
| 1 | 2 | 3 | | 4 | 5 | 7 | 9 |  |

***INSTRUCTION: GIVE A CARTE Q.15 TO AN INTERVIEWEE***

**Q.15 „Some people believe that whatever party people vote for, nothing will change. Others believe that who they vote for can change a lot of things. If you were to use the scale on this card, where ONE means that who people vote for won't change a thing, and FIVE means that who people vote for can change a lot of things, where would you place your opinion?“**

| WHO PEOPLE VOTE FOR WON’T CHANGE A THING | | |  | WHO PEOPLE VOTE FOR CAN CHANGE A LOT OF THINGS | | | REFUSAL | DK |  |
| --- | --- | --- | --- | --- | --- | --- | --- | --- | --- |
| 1 | 2 | 3 | | | 4 | 5 | 7 | 9 |  |

**Q.23 „How satisfied are you with the way democracy works in our country?**

| Very satisfied, ...................................................................................... | 1 |  |
| --- | --- | --- |
| rather satisfied, ...................................................................................... | 2 |  |
| rather dissatisfied, ................................................................................... | 3 |  |
| very dissatisfied.“.................................................................................. | 4 |  |
| REFUSAL ............................................................................... | 7 |  |
| DK ..................................................................................................... | 9 |  |

***INSTRUCTION: GIVE A CARTE Q.39 TO AN INTERVIEWEE***

**Q.39 „To what extent do you agree or disagree with each of the following statements“?**

| AGREE STRONGLY | agree | NEITHER AGREE NOR DISAGREE | disagree | disagree strongly | | REFUSAL | DK |  |
| --- | --- | --- | --- | --- | --- | --- | --- | --- |
| 1 | 2 | 3 | 4 | 5 | | 7 | 9 |  |
| a) Generally speaking, those we elect to public offices lose touch with citizens very quickly. | | | | | 1 2 3 4 5 7 9 | | |  |
| b) Politicians are only interested in winning citizens' votes, not their opinions. | | | | | 1 2 3 4 5 7 9 | | |  |
| c) You believe you would be as successful in public office as other people. | | | | | 1 2 3 4 5 7 9 | | |  |

| AGREE STRONGLY | AGREE | NEITHER AGREE NOR DISAGREE | DISAGREE | DISAGREE STRONGLY | REFUSAL | DK |  |
| --- | --- | --- | --- | --- | --- | --- | --- |
| 1 | 2 | 3 | 4 | 5 | 7 | 9 |  |
| d) You feel that you understand quite well the important political problems of our country. | | | | | 1 2 3 4 5 7 9 | |  |
| e) You don't think the government cares what people like you think. | | | | | 1 2 3 4 5 7 9 | |  |
| f) You consider yourself qualified enough to participate in politics." | | | | | 1 2 3 4 5 7 9 | |  |

**Q.40 „With which of the following statements you agree the most?**

| Democracy is better than any other way of governance...................... | 1 |  |
| --- | --- | --- |
| Under certain circumstances, the authoritative way of governance can be better than democratic ........ | 2 |  |
| For people like me, it does not matter whether we have democratic or undemocratic regime. ..................................... | 3 |  |
| REFUSAL ........................................................................................ | 7 |  |
| DK .............................................................................................................. | 9 |  |

**Q.41 "To what extent do you agree or disagree with the following statement?
"Democracy is the best form of government for a country like ours."**

| Agree strongly, ...................................................................................... | 1 |  |
| --- | --- | --- |
| agree, ............................................................................................. | 2 |  |
| neither agree, nor disagree, ....................................................................... | 3 |  |
| disagree, ......................................................................................... | 4 |  |
| disagree strongly.“ .................................................................................. | 5 |  |
| REFUSAL....................................................................................... | 7 |  |
| DK.............................................................................................................. | 9 |  |

***INSTRUCTION: GIVE A CARTE QCSN13 TO AN INTERVIEWEE***

**QCSN13 "To what extent do you agree or disagree with the following statement?**

| AGREE STRONGLY | AGREE | NEITHER AGREE, NOR DISAGREE | DISAGREE | DISAGREE STRONGLY | REFUSAL | | DK |  |
| --- | --- | --- | --- | --- | --- | --- | --- | --- |
| 1 | 2 | 3 | 4 | 5 | 7 | | 9 |  |
| a) If my favourite party doesn't stand a chance of winning, it's okay not to take part in the election. | | | | | | 1 2 3 4 5 7 9 | |  |
| b) Many elections are not important, so it's okay not to participate. | | | | | | 1 2 3 4 5 7 9 | |  |
| c) One vote has a negligible effect on the outcome of an election when a large number of people vote. | | | | | | 1 2 3 4 5 7 9 | |  |
| d) One should not vote unless one is interested in the results of the election." | | | | | | 1 2 3 4 5 7 9 | |  |

***INSTRUCTION: GIVE A CARTE OL.4 TO AN INTERVIEWEE***

**OL.4 "To what extent do you agree or disagree with the following statement?**

| AGREE STRONGLY | |  | DISAGREE STRONGLY | | | DK |  |
| --- | --- | --- | --- | --- | --- | --- | --- |
| 1 | 2 | 3 | 4 | 5 | | 9 |  |
| a) When you are among your friends and acquaintances, you often decide which topics are trending. | | | | | 1 2 3 4 5 9 | |  |
| b) Your friends and acquaintances often discuss topics you've raised. | | | | | 1 2 3 4 5 9 | |  |
| c) You usually succeed if you want to convince someone of something. | | | | | 1 2 3 4 5 9 | |  |
| d) It's easy for you to influence other people. | | | | | 1 2 3 4 5 9 | |  |
| e) Among your friends and acquaintances, you are often the one who has to approve important decisions. | | | | | 1 2 3 4 5 9 | |  |
| f) You are often asked to help friends and acquaintances make decisions. | | | | | 1 2 3 4 5 9 | |  |
| g) People around you often follow your advice. | | | | | 1 2 3 4 5 9 | |  |
| h) You seem to be considered a good source of tips and advice by your friends and acquaintances. | | | | | 1 2 3 4 5 9 | |  |
| i) You often use your powers of persuasion to reach agreement quickly during discussions." | | | | | 1 2 3 4 5 9 | |  |

**„And now a different topic.“**

EU.4 „How does your household handle the income it currently has?

| With great difficulty, ............................................................................ | 1 |  |
| --- | --- | --- |
| with difficulty, ...................................................................................... | 2 |  |
| rather with difficulty, ............................................................................. | 3 |  |
| rather easy, .................................................................................... | 4 |  |
| easy, ............................................................................................ | 5 |  |
| very easy.“ .................................................................................. | 6 |  |
| DK ................................................................................................ | 9 |  |

**EU.5 „Has your household ever found itself in a very difficult financial situation during the last 12 months?**

| Yes, ............................................................................................... | 1 |  |
| --- | --- | --- |
| no?“ ............................................................................................... | 2 |  |
| DK ............................................................................................... | 9 |  |

****EU.6 „Do you feel your household is rich or poor?****

| Very rich, .................................................................................. | 1 |  |
| --- | --- | --- |
| rather rich, ................................................................................... | 2 |  |
| neither rich, nor poor ....................................................................... | 3 |  |
| rather poor, .................................................................................... | 4 |  |
| very poor?“ .................................................................................. | 5 |  |
| DK ............................................................................................... | 9 |  |

*INSTRUCTION: GIVE A CARTE EU.23 TO AN INTERVIEWEE*

**EU.23 „Does your household's total income make it possible to**

| DEFINITELY YES | RATHER YES | RATHER NOT | DEFINITELY NOT | IRRELEVANT | DK |  |
| --- | --- | --- | --- | --- | --- | --- |
| 1 | 2 | 3 | 4 | 8 | 9 |  |
| a) meet basic household necessities - food, clothing and ordinary household goods, | | | | 1 2 3 4 8 9 | |  |
| b) satisfy the interests and hobbies of members of your household, | | | | 1 2 3 4 8 9 | |  |
| c) save money, | | | | 1 2 3 4 8 9 | |  |
| d) support your children's family or your parents, | | | | 1 2 3 4 8 9 | |  |
| e) buy healthier or better quality food, | | | | 1 2 3 4 8 9 | |  |
| f) holiday abroad regularly, | | | | 1 2 3 4 8 9 | |  |
| g) buy luxury goods, | | | | 1 2 3 4 8 9 | |  |
| h) pay for healthcare, medicines and medical supplies?“ | | | | 1 2 3 4 8 9 | |  |

***INSTRUCTION: GIVE A CARTE EU.16. TO AN INTERVIEWEE***

**EU.16 „How do you assess the situation in the Czech Republic with regard to the following areas?**

| VERY GOOD | RATHER GOOD | RATHER BAD | VERY BAD | | DK |  |
| --- | --- | --- | --- | --- | --- | --- |
| 1 | 2 | 3 | 4 | | 9 |  |
| a)Possibility to get an apartment, | | | | 1 2 3 4 9 | |  |
| b) financial possibilities for starting a family, to have children | | | | 1 2 3 4 9 | |  |
| c) old age security, | | | | 1 2 3 4 9 | |  |
| d) living conditions of the disabled, | | | | 1 2 3 4 9 | |  |
| e) opportunity to work, to be employed, | | | | 1 2 3 4 9 | |  |
| f) access to healthcare, | | | | 1 2 3 4 9 | |  |
| g)acces to education, | | | | 1 2 3 4 9 | |  |
| h)possibility to buy your own apartment or house.“ | | | | 1 2 3 4 9 | |  |

**„And now international issues“**

PM.11m "Are you interested in the latest developments on the refugee situation?

| Definitely yes, .......................................................................................... | 1 |  |
| --- | --- | --- |
| rather yes, ............................................................................................. | 2 |  |
| rather not, ............................................................................................... | 3 |  |
| definitely not.“ ........................................................................................ | 4 |  |
| DK ....................................................................................................... | 9 |  |

**PM.203 „Over the past period, mainly as a result of military conflicts, the European Union has faced an increased number of refugees. In your opinion, should the Czech Republic accept refugees from countries affected by military conflicts?**

| Yes, we should accept them and let them settle here, ........................ | 1 |  |
| --- | --- | --- |
| a yes, we should accept them until they are able to return to their country of origin., .......................................................................... | 2 |  |
| no, we should not accept refugees.“ ................................................. | 3 |  |
| DK ............................................................................................... | 9 |  |

**PM.204 „A large number of refugees from the Middle East and North Africa have come mostly to southern European countries that are unable to cope with such numbers of refugees. In your opinion, should the Czech Republic accept some of these refugees coming to the EU?**

| Definitely yes, ...................................................................................... | 1 |  |
| --- | --- | --- |
| rather yes,........................................................................................... | 2 |  |
| rather not, .......................................................................................... | 3 |  |
| definitely not?“ ................................................................................... | 4 |  |
| DK .................................................................................................... | 9 |  |

**PM.205 „The conflict in eastern Ukraine has also caused an increase in the number of Ukrainian refugees. Should the Czech Republic accept Ukrainians from war-affected areas?**

| Definitely yes, ...................................................................................... | 1 |  |
| --- | --- | --- |
| rather yes, .......................................................................................... | 2 |  |
| rather not, .......................................................................................... | 3 |  |
| definitely not?“ ................................................................................... | 4 |  |
| DK .................................................................................................... | 9 |  |

| **PM.214 „If the Czech Republic is in danger of losing money from European funds, should it accept refugees from Muslim countries?** | | |
| --- | --- | --- |
| Definitely yes,.................................................................................. | 1 |  |
| rather yes,...................................................................................... | 2 |  |
| rather not,...................................................................................... | 3 |  |
| definitely not.“................................................................................ | 4 |  |
| DK............................................................................................... | 9 |  |

***INSTRUCTION: GIVE A CARTE PM.189m TO AN INTERVIEWEE***

PM.189m „In your opinion, is the current situation around refugees a threat to

| definitely yes | rather yes | rather not | definitely not | | dk |  |  |
| --- | --- | --- | --- | --- | --- | --- | --- |
| 1 | 2 | 3 | 4 | | 9 |  |  |
| a) the security of the Czech Republic, | | | | 1 2 3 4 9 | | |  |
| b) European security | | | | 1 2 3 4 9 | | |  |
| c) peace in the world?“ | | | | 1 2 3 4 9 | | |  |

**„For the following questions (Q.32 to Q.35), please, if you do not remember the correct answer or you will not be sure, feel free to choose the answer "I do not know, I am not sure". This answer is more valuable to us than if you could guess the correct answer. "**

**Q.32 „Are Members of the Chamber of Deputies elected by a proportional or majority electoral system?**

| Proportional, ..................................................................................... | 1 |  |
| --- | --- | --- |
| majority, .................................................................................... | 2 |  |
| or don't you know, aren't you sure?"..................................................... | 9 |  |
| REFUSAL......................................................................... | 7 |  |

**Q.33 „Can you please say the name of the regional governor of your region (in the case of Prague, the incumbent mayor for the past four years)?"**

*REFUSAL= 97*

*DK = 99*

...........................................................................................

**Q.34 „Is the President of the European Commission elected by the citizens of the European Union?**

| Yes, ................................................................................................. | 1 |  |
| --- | --- | --- |
| no?“ .................................................................................................. | 2 |  |
| DK .................................................................. | 9 |  |
| REFUSAL .......................................................................... | 7 |  |

**Q.35 "Are the following statements true or false?**

| true | false | refusal | dk |  |
| --- | --- | --- | --- | --- |
| 1 | 2 | 7 | 9 |  |
| a) The Czech Republic was formally established in 1989. | | | 1 2 7 9 |  |
| b) A newly formed government must seek confidence in both the Chamber of Deputies and the Senate. | | | 1 2 7 9 |  |
| c) The European Union currently has 25 member states. | | | 1 2 7 9 |  |
| d) Members of regional councils are selected on the basis of the results of elections for regional councils. | | | 1 2 7 9 |  |
| e) Responsibility for the removal of domestic waste lies with the regional councils. | | | 1 2 7 9 |  |
| f) Canada is a permanent member of the United Nations Security Council. | | | 1 2 7 9 |  |
| g) Norway is not a member state of the European Union.“ | | | 1 2 7 9 |  |

**„And now, we will focus on the Internet.”**

**OM.31 „„ Do you use the Internet, that is, websites, emails or any other part of the Internet, whether from your computer, mobile phone, tablet, or other device?**

| Yes, ................................................................. | 1 | **=> OM.33a** |  |
| --- | --- | --- | --- |
| no?“ ................................................................ | 2 | **=> PZ.112 (str.13)** |  |
| DK ................................................................ | 9 | **=> PZ.112 (str.13)** |  |

***FILTER: ASK THOSE, WHO IN THE QUESTION OM.31 HAVE CHOSEN THE OPTION 1, THAT IS, THEY USE THE INTERNET***

**OM.33a „Do you use Facebook from home or from anywhere else?**

| More than once a week,.................................................................. | 1 |  |
| --- | --- | --- |
| less often, ..................................................................................... | 2 |  |
| or you do not use it at all?“ ............................................................. | 3 |  |
| DK ............................................................................................... | 9 |  |

***!!!ASK ALL!!!***

**PZ.112 „Could you tell how many books in total are in the household you live in? If you don't know, please try estimating their number by the fact that one meter contains about 40 books.**

| None, .............................................................................................. | 1 |  |
| --- | --- | --- |
| 1 – 2, ............................................................................................... | 2 |  |
| around 10, ........................................................................................ | 3 |  |
| around 20, ........................................................................................ | 4 |  |
| around 50, ....................................................................................... | 5 |  |
| around 100, .................................................................................... | 6 |  |
| around 200, ..................................................................................... | 7 |  |
| around 500, ..................................................................................... | 8 |  |
| more than 1000.“ ............................................................................. | 9 |  |
| DK..................................................................... | 99 |  |

***INSTRUCTION: GIVE A CARTE PV. 1A TO AN INTERVIEWEE.***

**PV.1a „Please tell me, on a scale of 1 to 10, how likely it is that you would go voting, if next week there were elections to the Chamber of Deputies? When 1 represents someone, who definitely will not vote, and 10 represents someone who will definitely vote, where on this scale from 1 to 10 would you rank yourself?“**

*NO VOTING RIGHTS= 98*

| definitely won’t go | | | | | definitely will go | | | | | | DK |  |
| --- | --- | --- | --- | --- | --- | --- | --- | --- | --- | --- | --- | --- |
| 1 | 2 | 3 | 4 | 5 | | 6 | 7 | 8 | 9 | 10 | 99 |  |

**„To conclude, let s pose only a few questions necessary for the statistical processing of research results.“**

***INSTRUCTION: GIVE A CARTE IDE.5c TO AN INTERVIEWEE.***

**IDE.5c „Try to put into one of the groups.“**

| *CODES1,2,4*  *CODE 3*  *CODES 5-17* | => continue on ***EU.179***  => continue on ***EU.178***  => continue on ***IDE.5b*** |  |  |
| --- | --- | --- | --- |
|  |  | REWRITE ONLY A DIGITAL CODE: |  |

***FILTER: ONLY FOR ECONOMICALLY ACTIVE (CODES FROM 5 TO 17 IN QUESTION IDE.5c) INSTRUCTION: GIVE A CARTE IDE.5b TO AN INTERVIEWE***

**IDE.5b „In what industry do you work?“**

|  | => *continue on* ***EU.179*** | REWRITE ONLY A DIGITAL CODE: |  |
| --- | --- | --- | --- |

¨***FILTER: ONLY FOR THE UNEMPLOYED (CODE 3 IN QUESTION IDE.5c)***

**EU.178 „For how long have you been unemployed?”**

*LESS THAN A MONTH = 997*

| *REFUSAL = 998*  *DK = 999* | WRITE THE NUMBER OF MONTHS: |  |
| --- | --- | --- |

***!!! ASK ALL !!!***

**EU.179 „Were you unemployed for more than 6 months?**

| YES | NO | DK | |  |
| --- | --- | --- | --- | --- |
| 1 | 2 | 9 | |  |
| a) whenever in your life, | | | 1 2 9 |  |
| b) during last 5 years?“ | | | 1 2 9 |  |

****IDE.2 „How old are you?"****

|  |  |
| --- | --- |

***INSTRUCTION: GIVE A CARTE IDE.6b TO AN INTERVIEWEE***

**IDE.6b „What is the highest educational level that you have attained?“**

| NUMERIC CODE: |  |
| --- | --- |

***INSTRUCTION: GIVE A CARTE PO.2 TO AN INTERVIEWEE***

**PO.2 „In political matters, people talk of „the left“ and „the right.“ How would you place your views on this scale, generally speaking?“**

*DK = 99*

| LEFT | | | | | | RIGHT | | | | | |  |  |
| --- | --- | --- | --- | --- | --- | --- | --- | --- | --- | --- | --- | --- | --- |
| L | L | L | L | L | 0 | | R | R | R | R | R |  |  |
| a | b | c | d | e | f | | g | h | i | j | k |  |  |
| 1 | 2 | 3 | 4 | 5 | 6 | | 7 | 8 | 9 | 10 | 11 |  |  |

**PV.182 „Did you vote in the last general elections in October 2017?**

| Yes, ...................................................................... | 1 | **=> PV.183** |  |
| --- | --- | --- | --- |
| no, ........................................................................ | 2 | **=> PV.153a** |  |
| not eligible to vote.“ ........................................ | 3 | **=> PV.153a** |  |
| DK ...................................................................... | 9 | **=> PV.153a** |  |

| ***FILTER: ASK THOSE, WHO IN THE QUESTION PV.182 ANSWERED YES (VARIANT 1)*** | | |
| --- | --- | --- |
| **PV.183 „Which party did you vote for in that elections?“** | | |
| ANO 2011,............................................................................................. | 1 |  |
| Czech Pirate Party (Piráti),.................................................................. | 2 |  |
| Czech Social Democratic Party (ČSSD),.............................................. | 3 |  |
| Christian and Democratic Union – Czechoslovak People’s Party (KDU-ČSL),.. | 4 |  |
| Communist Party of Bohemia and Moravia (KSČM),....................................... | 5 |  |
| Civic Democratic Party (ODS),....................................................... | 6 |  |
| Freedom and Direct Democracy– Tomio Okamura (SPD),............................... | 7 |  |
| Mayors and the Independent (STAN),.......................................................... | 8 |  |
| Party of Free Citizens (Svobodní),....................................................... | 9 |  |
| Green Party (Zelení),......................................................................... | 10 |  |
| TOP 09,.................................................................................................. | 11 |  |
| Other.“............................................................................................ | 12 |  |
| DK...................................................................................................... | 99 |  |
| Threw in an empty envelope.................................................................... | 96 |  |

***!!! ask all!!!***

**PV.153a „On 26 and 27 January 2018, the second round of direct elections of the President of the Czech Republic took place. Were you voting?**

| Yes, ......................................................................... | 1 | **=> PV.159a** |  |
| --- | --- | --- | --- |
| no.“ .......................................................................... | 2 | **=> IDE.10a (str.15)** |  |
| Not eligible to vote .............................................. | 8 | **=> IDE.10a (str.15)** |  |
| DK.......................................................................... | 9 | **=> IDE.10a (str.15)** |  |

***FILTER: ASK THE FOLLOWING QUESTION THOSE, WHO IN QUESTION PV.153a HAVE CHOSEN AN OPTION 1 (THAT IS, THEY TOOK PART IN THE SECOND ROUND OF PRESIDENTIAL ELECTION).***

**PV.159a „Who did you vote in the second round?**

| Miloš Zeman, ........................................................................................ | 1 |  |
| --- | --- | --- |
| Jiří Drahoš.“ ........................................................................................ | 2 |  |
| DK ........................................................................................................ | 9 |  |

***!!! ask all !!!***

**IDE.10a „What is your total net monthly income, i.e. only your person's income? If you are not sure, please estimate at least an approximate amount.“**

| *REFUSAL= 8*  *NO INCOME = 7*  GIVE A NUMBER: |  |  |  |  |  |  | Kč |
| --- | --- | --- | --- | --- | --- | --- | --- |

**IDE.10 „And what is the usual net monthly income of your entire household, i.e. when you add up the income of all the household members? If you are not sure, please estimate at least an approximate amount.“**

| *REFUSAL= 8*  *DK = 9*  GIVE A NUMBER: |  |  |  |  |  |  | Kč |
| --- | --- | --- | --- | --- | --- | --- | --- |

IDE.3a „Are you

| single, ........................................................................... | 1 |  |
| --- | --- | --- |
| married (or live in a registered partnership), ......................... | 2 |  |
| divorced, ......................................................................... | 3 |  |
| widow/widower.“ ................................................................................. | 4 |  |
| DK .................................................................................................. | 9 |  |

IDE.3b "Do you live in your household with your spouse or permanent partner?"

| YES ................................................................................................... | 1 |  |
| --- | --- | --- |
| NO ..................................................................................................... | 2 |  |

IDE.13 „How many members, including you, do your household have? Remember to include yourself in the total..“

| NUMBER OF PEOPLE: |  |
| --- | --- |

**IDE.12 „Do you have dependent children? If so, how much? (We're not asking about household children, we're asking about your dependent children.)**

| One child, ...................................................................... | 1 |  |
| --- | --- | --- |
| two children, ......................................................................... | 2 |  |
| three children, ........................................................................... | 3 |  |
| four or more children, ....................................................... | 4 |  |
| has no child.“ ......................................................... | 5 |  |

**IDE.57 „How many members of your household is economically active (have a paid job)? If you are also economically active, please include yourself, as well.“**

| *NO ECONOMICALLY ACTIVE MEMBER*  *IN A HOUSEHOLD = 98* | GIVE A NUMBER: |  |
| --- | --- | --- |

***INSTRUCTION: GIVE A CARTE IDE.7 TO AN INTERVIEWEE***

**IDE.7** **„To what church or religious community do you belong to?**

| *DK = 9* | WRITE ONLY A DIGITAL CODE: |  |
| --- | --- | --- |

***INSTRUCTION: GIVE A CARTE IDE.19 TO AN INTERVIEWEE***

**IDE.19 „When you look at the IDE.19 carte, how would you mark the place you live in?“**

| *DK = 9* | WRITE ONLY A DIGITAL CODE: |  |
| --- | --- | --- |

**“On behalf of Public Opinion Research Center, I would like to thank you for the interview.”**

**QUESTIONS FOR THE INTERVIEWERS (ANSWER DIRECTLY AFTER THE FINISHED INTERVIEW)**

| **IDE.8 AN INTERVIEWEE IS:** | A MAN = 1  A WOMAN = 2 |  |  |
| --- | --- | --- | --- |

| **IDE.9 AN INTERVIEWEE LIVES IN A DISTRCIT NUMBER** |  |  |  |
| --- | --- | --- | --- |

| **IDE.72 TIME AT THE END OF THE INTERVIEW:** |  |  | **:** |  |  |
| --- | --- | --- | --- | --- | --- |

| **IDE.55 5 HOW MANY PEOPLE REFUSED TO TAKE PART IN THIS PARTICULAR INTERVIEW (PLEASE INCLUDE ONLY THOSE WHO WERE SUITABLE FOR THE DESIGNED SAMPLE** | |  |
| --- | --- | --- |
|  | *NOBODY REFUSED = 98* |  |

IDE.56 PLACE OF THE INTERVIEW

| Interviewee’s home, ...................................................................... | 1 |  |
| --- | --- | --- |
| Interviewer’s home, ............................................................................ | 2 |  |
| Work place (interviewer’s or interviewee’s), ........................................... | 3 |  |
| Public place (street, park, school, parking place) ........................ | 4 |  |
| Waiting room (railway station, at the doctor’s, etc.),................................. | 5 |  |
| Restaurant, ............................................................................ | 6 |  |
| Other place. Please specify: .................................................... | 7 |  |
|  | | |
| IDE.70 AN INTERVIEWEE LIVES IN A MUNICIPALITY / TOWN.……………………………………………… | | |

| INTERVIWER’S EVIDENCE NUMBER |  |  |  | |  |  | |  |  |  |  |  |
| --- | --- | --- | --- | --- | --- | --- | --- | --- | --- | --- | --- | --- |
|  | region | | | VSO | | | PERSONAL NUMBER | | | | | |

| INTERVIEWER’S SURNAME: | ............................................................................ |
| --- | --- |

**I hereby confirm that I have chosen an interviewee and conducted the interview according to the Public Opinion Research Center instruction and that I have stuck to the Ethical Code of the Interviewer.**

| SIGNATURE: | .……………………………………………………….................................... |
| --- | --- |
| DARE: | ………………………………………………………..................................... |

| **PLEASE CHECK: 1. ALL QUESTIONS CONTAIN INTERVIEWEE’S ANSWERS THERE IS A CIRCLE AROUND THE PROPER CODE OF THE ANSWER.**  **2. ALL THE SQUARES ARE FILLED IN WITH NUMBERS.**  **3. IF AN INTERVIEWEE DID NOT ANSWER SOME QUESTIONS (E.G. FILTER), THERE IS “0” NUMBERIN A SQUARE.** |
| --- |

| **EVALUATION OF THE QUESTIONNAIRE (PUBLIC OPINION RESEARCH CENTER TO FILL IN)** |  |
| --- | --- |
